# Knowledge, attitudes, and practices towards vector-borne diseases in changing climate in Finland

**DOI:** 10.1017/S0950268824001468

**Published:** 2025-01-15

**Authors:** Henna Mäkelä, Timothée Dub, J. Pekka Nuorti, Jussi Sane

**Affiliations:** 1 Department of Health Security, Infectious Diseases Control and Vaccination Unit, Finnish Institute for Health and Welfare (THL)Helsinki, Finland; 2 Health Sciences Unit, Faculty of Social Sciences, Tampere University, Tampere, Finland

**Keywords:** attitudes, and practices, KAP, knowledge, Lyme borreliosis, tick-borne encephalitis, vector-borne diseases

## Abstract

With climate change, the geographic distribution of some VBDs has expanded, highlighting the need for adaptation, and managing the risks associated with emergence in new areas. We conducted a questionnaire survey on the knowledge, attitudes, and practices (KAP) about vector-borne diseases (VBDs) among sample of Finnish residents. The questions were scored and the level of KAP was determined based on scoring as poor, fair, good, or excellent. Binary logistic regression analysis was used to evaluate the associations of different KAP levels with sex, age, education, and possible previous VPD infection. We received 491/1995 (25%) responses across the country and detected generally good knowledge, but only fair practices towards VBDs. Sex and age of the respondents were most often significantly associated with the level of KAP (*P* > 0.05). Despite the generally good knowledge, we detected major gaps, especially regarding the distinction of tick-borne encephalitis and Lyme borreliosis (LB), risk of disease, and protective measures. Additionally, many respondents thought the vaccination protects against LB or tick bites. This calls for awareness raising on disease risk and prevention measures. With increasing cases and the effects of climate change, surveillance of VBDs communication to the general public should be strengthened.

## Introduction

Over the past decades, the incidence of many vector-borne diseases (VBDs) has increased in Europe and the geographic areas for VBDs have expanded in line with ticks and other vectors spreading northwards and to higher altitudes [1–3]. VBDs are considered as emerging or re-emerging diseases, as they (re)occur in regions that were previously free from these diseases and differ from their previous distribution patterns [4]. As vector species are spending most of their life-cycle independent from the host animal, environmental factors such as temperature and humidity are playing a key role in their prevalence and distribution [2, 5].

Climate change has ongoing effects on the epidemiology of VBDs [6, 7]. In the Arctic regions, climate change is expected to cause much higher temperature rises than most other regions [8]. Climate change affects the transmission and the distribution of VBDs through multiple complex pathways [8]. In addition to the effects on the pathogen and vector species, climate change also influences the behaviours of human and non-human hosts, changes in environment, sociodemographic factors, and the healthcare infrastructure [7].

We studied four VBDs of public health importance in Finland, including two tick-borne diseases (TBDs), tick-borne encephalitis (TBE), and Lyme borreliosis (LB), and two mosquito-borne diseases (MBDs), Pogosta disease and tularaemia. In Finland, the annual incidence of clinically diagnosed cases of both LB caused by *Borrelia burgdorferi* and TBE as well as tick abundance have increased in the past 20 years [9, 10]. However, a seroprevalence study using samples from 1960s and 1970s indicated that exposure to LB was considerably more common than in 2011 [11]. Additionally, the geographical distribution of *Ixodes ricinus* and *Ixodes persulcatus* have increased over the past decades in Fennoscandia, that is, Finland, Sweden, and Russia, particularly in inner land areas [2, 3, 12–14]. Although the vectors have been found all around the country except northern Lapland, the risk for TBE varies. The high-incidence areas are small and limited geographically. Previously, the high-incidence areas have been in the southwestern coast and archipelago of Finland, but currently the disease has been detected also elsewhere in the country.

An important step in public health prevention and control is to assess the population’s knowledge, attitudes, and practices (KAP) towards VBDs so that public health organizations can provide timely, accurate, and evidence-based risk communication. We assessed KAP to detect potential knowledge gaps and misbeliefs.

## Methods

### Study design and sample

The study sample was collected by the Finnish Digital and Population Data Service Agency (DVV), and they were requested to sample adult residents ≥18 years from each hospital district (HD) corresponding to the age and sex distribution of the population in the country. As Finland has two official languages (Finnish and Swedish), the respondents´ first language was one of the variables that were requested from the DVV. There were also substantial differences in population size between the districts, and we therefore invited the same number of individuals from all districts with the aim to enrol participants from the whole country. The final sample size was 1995 including 95 residents from each of the 21 HDs. The participants were invited to send their response during the study period August to October in 2020. The KAP survey questionnaire was mailed to the study participants, and the responses were anonymous.

### Questionnaire

The questionnaire in Finnish and Swedish was mailed to the selected sample without a follow-up letter. The language and wording chosen for the questionnaire were aimed to be as simple and understandable as possible, and thus, for example, the term ‘Lyme borreliosis’ was used across the questionnaire instead of *B. burgdorferi* and Erythema migrans was replaced by term ‘a ring-like rash’. In addition to the questions related to diseases and vectors, the questionnaire also included some questions related to climate change. A link to the online questionnaire was provided and encouraged to use as the primary option. The questionnaire consisted of four different sections: knowledge, attitudes, practices, and background information with a total of 28 questions including four matrices (Supplementary Material, Appendix 1). We used a five-point Likert-type scale in the questionnaire. For knowledge and attitude-related questions, the options were as follows: ‘Strongly agree’, ‘Agree’, ‘Neither disagree nor agree’, ‘Disagree’, and ‘Strongly disagree’. For practice-related questions, the options were as follows: ‘Always’, ‘Often’, ‘Sometimes’, ‘Rarely’, and ‘Never’. For the analysis, these categories were further merged. As a result, we had three different categories: ‘Agree’ (combining ‘Strongly agree’ and ‘Agree’), ‘Neither disagree nor agree’, and ‘Disagree’. For practice-related questions, the categories were as follows: ‘Often’ or ‘Always’, ‘Sometimes’, and ‘Never’ or ‘Rarely’. The questions and statements were selected to gain comprehensive information on respondents’ KAP, and not all responses to statements could be categorized into ‘factual’ or ‘incorrect’, and such questions were thus excluded from the KAP scoring (Supplementary Material, Appendix 2).

### Statistical analysis

Differences in proportions between the respondents and the original sample in terms of sex, age, and area were assessed using the chi-square test.

We calculated KAP scores for each respondent. Scoring of appropriate responses relied on the current evidence-based knowledge. Not all statements and questions were scored (Supplementary Material, Appendix 2). In the knowledge section, the answers in line with the current literature were considered appropriate and were scored with one point and the incorrect answers with zero points. This was done both with questions with options and with the matrix. With all the other matrices, we scored the right answer with two points (strongly agree/strongly disagree) and the answers of agree/disagree with one point depending on a question. We designated the wrong answers as zero points (Supplementary Material, Appendix 2). Based on the scores of different categories, we further defined the level of KAP as poor, fair, good, or excellent. This categorization was based on percentages of points received: with ≤25% points, the level was categorized as poor, with 26–50% as fair, 51–75% good and >75% as excellent. We calculated this individually for each category with TBDs and MBDs.

Finally, we fitted binary logistic regression models to determine the factors associated with the levels of KAP. We conducted single variable analysis adjusted for age, sex, and HD before conducting multivariable analyses. Variables included in the model were education level, TBE vaccination status, personal history of infection with family and friends, native language, and activity level which was based on reported times spent outdoors weekly or monthly. All these factors were self-reported through the questionnaire. We used a *P* value of 0.25 as the screening criterion for the selection of variables for the multivariable analyses. Statistical significance was considered at 5% level.

We performed the regression analyses separately for mosquito-, tick-, and general VBDs. The statistical analysis was performed using R 3.6.0 software.

### Ethical approval

This anonymized, population-based survey did not require specific ethics approval, according to the ethics guidelines of the Finnish Institute for Health and Welfare and the provisions of the Communicable Diseases Act.

## Results

**Figure 1.**
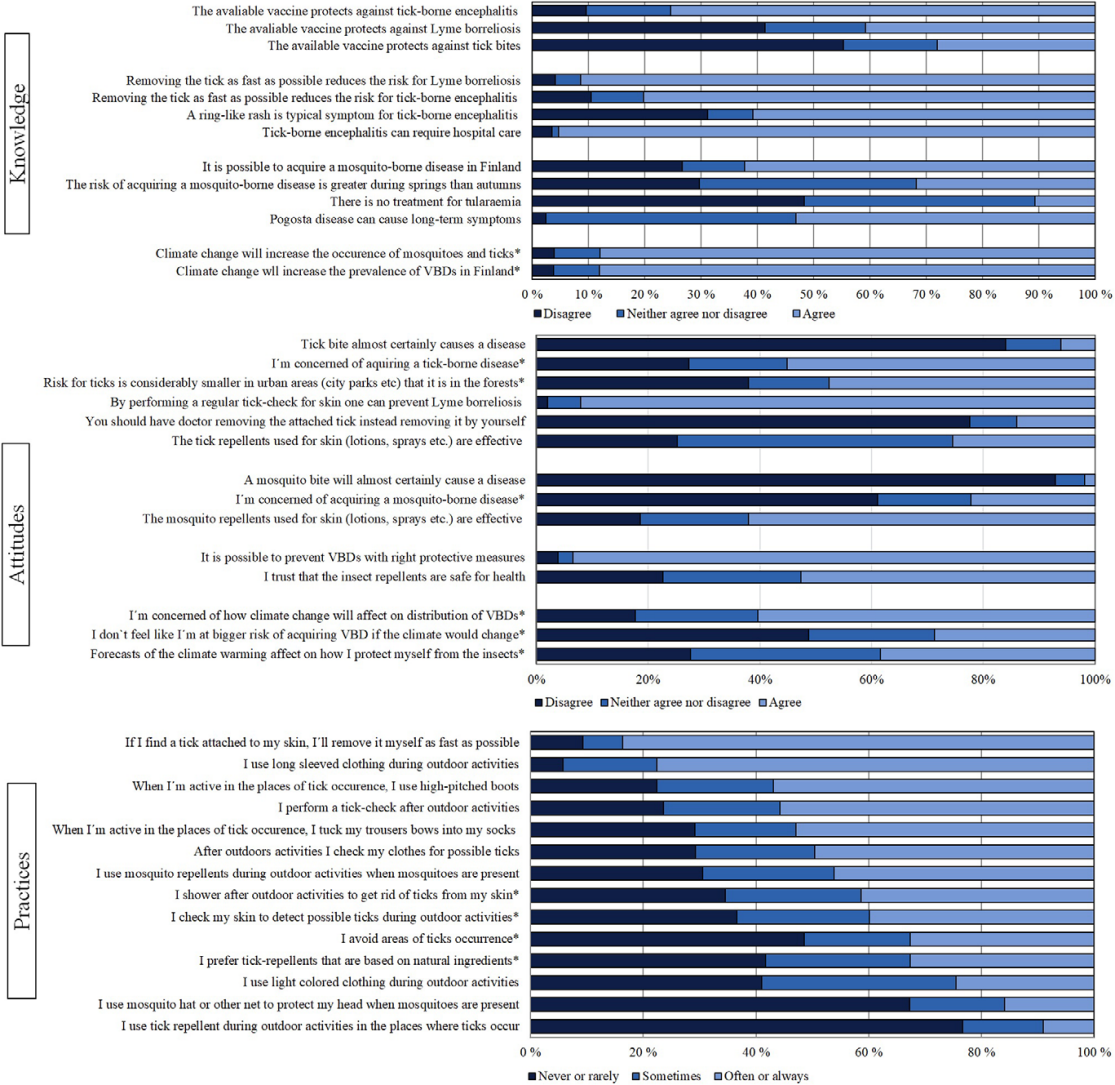
Responses to knowledge, attitudes and practices questions. The histogram shows the distribution of three possible answers. Statements marked with * were not included to KAP-scoring.

**Figure 2.**
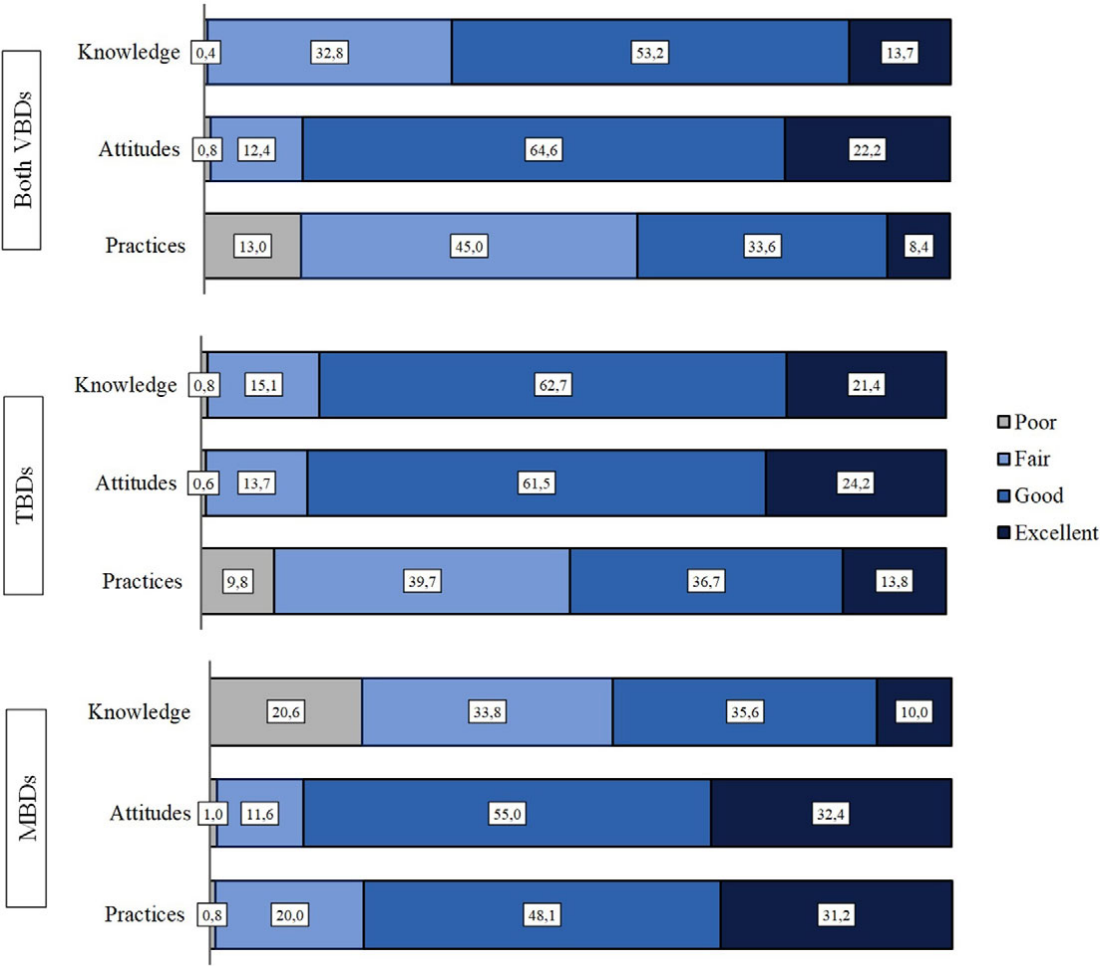
KAP-levels regarding vector-borne diseases, tick-borne diseases and mosquito-borne diseases.

We received a total of 491 responses (24.6%) in September to October 2020. The response rate varied between different regions from 16% (South Ostrobothnia) to 40% (Åland). The responses to all statements are shown in Figure 1 and the results of calculated KAP scores are shown in Figure 2.

### Demographics

Fifty-six per cent of the respondents were female and 91% Finnish speaking. The proportions of Swedish and Finnish speakers and men and women were similar among respondents and non-responders. However, the median age of respondents was 63 years, and they were significantly older compared to non-respondents (median, 63 years vs. 45.5 years, *P* < 0.05). Demographics are shown in Table 1 and further characteristics of the respondents are shown in Table 2.

**Table 1.** Demographics and general information about the respondents

Characteristics	*n* = 491
	*n* (%)
Sex	
Female	273 (55.6)
Language	
Finnish	442 (90.0)
Swedish	49 (10.0)
Highest level of education	
Comprehensive school	104 (21.1)
Upper secondary school/vocational school	195 (39.6)
Bachelor degree/university of applied sciences	118 (23.9)
Masters degree	63 (12.8)
Doctorate	7 (1.4)
Activity	
Daily	136 (27.7)
3–4 times a week	141 (28.7)
About once a week	92 (18.7)
A few times a month	40 (8.1)
About once a month	3 (0.6)
Less frequently	21 (4.3)
Never	4 (0.8)
Activity varies	51 (10.4)
Pet owner	
Either cat or dog	160 (32.6)
Both	20 (4.1)
Found ticks from the pet (*n* = 188)	
Yes	147 (81.7)

**Table 2.** Information related to TBE vaccination, history of infection, and information sources reported by the respondents

Characteristics	*n* = 491
	*n* (%)
TBE vaccination	
Vaccinated	102 (20.8)
Reason for TBE vaccination	
Living in the risk-area	46 (27.4)
Spending a lot of time in the risk area/owning a summerhouse in the area	39 (23.2)
Being active in the risk areas	57 (33.9)
Other reason	26 (15.5)
Reason for not having TBE vaccination	
No need for the vaccination – not active nor living in the risk area	223 (53.5)
Price of the vaccination	43 (10.3)
Vaccination was not recommended by doctor or nurse	47 (11.3)
Problems with the vaccination schedule	15 (3.6)
Other reason	89 (21.3)
History of infection	
Diagnosed with VBD	46 (9.4)
Friend or family diagnosed with VBD	81 (16.5)
Information sources of VBDs	
Newspapers	207 (42.2)
Webpage of public health institute	229 (46.6)
Somewhere else form the internet	165 (33.6)
Social media (Facebook, Twitter, Instagram, etc.)	58 (11.8)
Work or school	46 (9.4)
Vaccination campaigns	138 (28.1)
Healthcare professional	195 (39.7)
Family and friends	103 (21.0)
Own, experience-based knowledge	72 (14.7)
Somewhere else	21 (4.3)

### Climate change

Most respondents agreed on the statement that climate change increases the prevalence of VBDs in Finland (88%, 423/480) and that climate change will increase the occurrence of mosquitoes and ticks (88%, 242/482). When asked if the respondents were concerned about how climate change will affect the distribution of VBDs, 60% (294/487) expressed concern (Figure 1). However, only half felt like they would have an increased risk of acquiring VBD infection if the climate were to change (49%, 237/487). Finally, when asked if the forecasts of climate change and, that is, warmer winters, would affect how respondents protect themselves from insects, only 39% (187/486) agreed.

### Knowledge

The general knowledge on VBDs was good among the respondents (53%). The good knowledge related to TBDs was higher (63%), whereas the knowledge on MBDs was good only for 34% of the respondents and up to 21% having poor knowledge (Figure 2).

Almost all the respondents had heard of TBDs, but MBDs were unknown to 70% (344/496) who reported of having heard of tularaemia and only 33% (163/496) of Pogosta disease. Half of the respondents knew TBE is caused by a virus (52%, 251/482). When asked about the transmission of the diseases, respondents were more familiar with TBDs than MBDs with 95% (463/490) knowing that borreliosis is transmitted by ticks and only 27% (131/496) knowing that tularaemia is mainly associated with transmission by insects in Finland and 33.8% (176/496) knowing that Pogosta disease is transmitted by mosquitoes.

Only one third of the respondents knew that TBE can be transmitted by all tick stages from larvae to nymph to adult tick while the majority (60%) thought that it is only adult ticks that transmit the disease. When assessing knowledge about the risk of TBE by asking for the approximate percentage of ticks carrying the TBE virus in high-risk areas, many respondents either did not know the percentage (28%, 138/496), indicated 10–20% (23%, 114/496), or 4–8% (22%, 108/496). Only 17% (85/496) of respondents chose the correct option of 1–2%.

When asked about the available vaccination, 75% (362/480) knew that it provides protection against TBE. However, 41% (14/475) of respondents thought it provides protection from LB and 28% (135/481) that it protects against tick bites. In addition, 61% (293/482) responded the ring-like rash being a symptom of TBE (Figure 1). Majority (95%, 460/483) agreed that TBE can require hospital care. In addition, 80% of respondents (445/487) agreed on the statements that removing tick as fast as possible reduces the risk for TBE.

Age and activity of the respondents were significantly associated with the level of TBD knowledge (Supplementary Material). Compared to the age group 18–29 years, all other groups had slightly worse knowledge, especially respondents aged 70–79 years (OR = 0.22, 95% CI: 0.01–1.53)). Age, language, and activity were associated with the level of MBD knowledge as those who were active daily were more likely to have better knowledge than those who were less active in the nature, older respondents were more likely to have a good level of knowledge compared to young respondents and Swedish-speaking respondents were 70% more likely to have poor knowledge regarding MBDs compared to Finnish speakers (Table 3).

**Table 3.** Univariate and multivariable analysis of factors associated with good knowledge regarding mosquito-borne diseases

	*n*	Univariate analysis	*P*	Multivariable analysis	*P*
		OR (95 Cl)		OR (95 Cl)	
Sex		*Adjusted for sex, age, and hospital district*			
Male	218			1.10 (0.71–1.60)	
Female	273			1	
Age					
18–29	48	*Adjusted for sex, age, and hospital district*		1	
30–39	53			1.75 (0.64–5.09)	
40–49	37			2.79 (0.95–8.56)	
50–59	80			4.62 (1.87–12.51)	8.782e–07
60–69	115			7. 68 (3.20–20.33)	
70–79	119			6.79 (2.86–17.82)	
80+	39			3.53 (1.24—10.65)	
Hospital district		*Adjusted for sex, age, and hospital district*			
Ahvenanmaa	27			0.62 (0.11–3.32)	
Etelä-Karjala	23			1.64 (0.45–6.15)	
Etelä-Pohjanmaa	15			2.45 (0.57–11.30)	
Etelä-Savo	19			1.27 (0.34–4.88)	
HUS	22			1	
Itä-Savo	27			2.31 (0.64–8.90)	
Kainuu	22			1.17 (0.32–4.36)	
Kanta-Häme	33			0.92 (0.28–3.07)	
Keski-Pohjanmaa	26			1.46 (0.40–5.41)	
Keski-Suomi	27			1.03 (0.29–3.72)	
Kymenlaakso	23			0.68 (0.18–2.49)	0.61
Lappi	22			0.52 (0.12–2.14)	
Länsi-Pohja	17			0.86 (0.23–3.23)	
Pirkanmaa	26			1.18 (0.32–4.46)	
Pohjois-Karjala	22			1.12 (0.30–4.20)	
Pohjois-Pohjanmaa	18			0.72 (0.18–2.85)	
Pohjois-Savo	26			2.44 (0.70–8.88)	
Päijät-Häme	18			1.19 (0.30–4.80)	
Satakunta	24			0.57 (0.15–2.14)	
Vaasa	21			1.40 (0.32–6.34)	
Varsinais-Suomi	35			0.97 (0.29–3.31)	
Highest education				Not in the model	
Comprehensive	104	0.56 (0.29–1.05)			
Secondary high school or vocational school	195	0.67 (0.40–1.12)	066559		
Bachelor or university of applied sciences	118	1			
Masters of doctorate	70	0.65 (0.33–1.27)			
TBE vaccination status				Not in the model	
Not vaccinated	379	1	Ref		
Vaccinated	102	0.76 (0.44–1.31)	0.33		
Personal history of VBD infection				Not in the model	
No	433	1	Ref		
Yes	46	1.44 (0.69–3.08)	0.34		
History of VBD infection with family and friends				Not in the model	
No	398	1	Ref		
Yes	81	1.41 (0.79–2.54)	0.25		
Language					
Finnish	442	1	Ref	1	
Swedish	49	0.25 (0.08–0.71)	0.01	0.27 (0.08–0.79)	0.00006
Activity					
Daily	136	1.21 (0.71–2.06)		1.25 (0.73–2.14)	
3–4 times a week	141	1		1	
About once a week	92	0.67 (0.36–1.21)		0.68 (0.37–1.23)	
A few times a month	40	0.53(0.23–1.17)		0.53 (0.23–1.19)	
About once a month	3	0.41 (0.02–5.59)		0.41 (0.02–5.62)	
Less frequently	21	0.73 (0.25–2.02)		0.79 (0.27–2.20)	
Never	4	4.44 (0.49–98.07)		2.43 (0.36–78.2)	
Acitivity varies	51	0.84 (0.41–1.72)		0.82 (0.40–1.67)	

### Attitudes

Attitudes were overall good (65%) with VBDs and only a small difference was observed between TBDs and MBDs, where 62% had good attitudes towards VBDs and 55% towards MBDs (Figure 2).

Most of the respondents agreed that with right protective measures VBDs can be prevented (93%, 456/488) and 92% (449/484) that by a regular tick check one can prevent LB (Figure 1). What it comes to repellents, 62% (300/483) thought that mosquito repellents for skin are effective, but only 26% (123/483) though so with tick repellents (Figure 1).

We found that with TBDs, sex and language were significantly associated with attitudes. Men (OR = 0.47, 95% CI: 0.31–0.71) and Swedish speakers (OR = 0.25, 95% CI: 0.07–0.72) were more likely to have attitudes that were considered poor compared with women and Finnish speakers (Supplementary Material). With MBDs, men were more likely to have attitudes considered poor compared with women (VBDs: OR = 0.44, 95% CI: 0.28–0.69; MBDs: OR = 0.71, 95% CI: 0.19–2.48) (Supplementary Material).

### Practices

Altogether, the practices were only fair with VBDs (45%). Nevertheless, the practices were significantly better with MBDs than TBDs as 48% were observed having good practices, 32% excellent practices, and only less than 1% poor practices. With the TBDs, the numbers were 37%, 14%, and 10%, respectively.

Respondents (229/491, 47%) reported seeking information about VBDs from the webpage of a public health agency and 42% (207/491) from newspapers, followed by healthcare professional (40%, 195/491), and somewhere else from the internet (34%, 165/491). Twenty-eight percent (138/491) said that they received the needed information from vaccination campaigns and 21% (103/491) from family and friends. Most of the respondents (64%, 314/491) indicated they used skin sprays as insect repellents. Almost 40 % (39%, 192/491) used mosquito smoke for repelling mosquitoes and 28% (138/491) stated that they are using mosquito repellent that works with gas. Up to 17% (85/491) said that they are not using any repellents.

Most used preventative measure was removing an attached tick as quickly as possible from the skin (84%, 394/471), followed by using long-sleeved clothing (78%, 374/482), using high-pitched boots during outdoor activities (57%, 272/478) and performing tick check after outdoor activities (56%, 264/474). The least used preventative measure was using tick repellents (9%, 43/476) and using mosquito hat or net for head protection (16%, 76/477) (Figure 1).

We found that with TBDs, sex and age were significantly associated with the level of practices (Table 4). With MBDs, sex and personal history of infection were observed to be significant variables related to the level of practices, with males and those having had infection being more likely to have poor practices (Supplementary Material).

**Table 4.** Univariate and multivariable analysis of factors associated with good practices regarding tick-borne diseases

	*n*	Univariate analysis	*P*	Multivariable analysis	*P*
		OR (95 CI)		OR (95 CI)	
Sex		*Adjusted for sex, age, and hospital district*			
Male	218			0.22 (0.14–0.36)	0.00000003
Female	273			1	
Age					
18–29	48	*Adjusted for sex, age, and hospital district*		1	
30–39	53			0.57 (0.15–1.93)	
40–49	37			2.13 (0.65–7.02)	
50–59	80			2.16 (0.85–5.84)	0.0001
60–69	115			3.24 (1.34–8.47)	
70–79	119			4.21 (1.73–11.08)	
80+	39			2.86 (0.96–8.88)	
Hospital district		*Adjusted for sex, age, and hospital district*			
Ahvenanmaa	27			0.56 (0.05–4.72)	
Etelä-Karjala	23			2.17 (0.52–9.80)	
Etelä-Pohjanmaa	15			1.76 (0.31–9.87)	
Etelä-Savo	19			1.42 (0.30–6.89)	
HUS	22			1	
Itä-Savo	27			1.53 (0.37–6.71)	
Kainuu	22			1.39 (0.33–6.24)	
Kanta-Häme	33			1.29 (0.34–5.31)	
Keski-Pohjanmaa	26			1.56 (0.36–7.12)	
Keski-Suomi	27			1.45 (0.34–6.40)	
Kymenlaakso	23			3.82 (0.94–16.94)	0.22
Lappi	22			1.39 (0.31–6.51)	
Länsi-Pohja	17			1.90 (0.37–9.69)	
Pirkanmaa	26			0.35 (0.04–2.13)	
Pohjois-Karjala	22			1.96 (0.46–9.02)	
Pohjois-Pohjanmaa	18			2.82 (0.61–13.81)	
Pohjois-Savo	26			1.87 (0.46–8.10)	
Päijät-Häme	18			0.78 (0.12–4.39)	
Satakunta	24			1.98 (0.47–8.84)	
Vaasa	21			3.08 (0.60–16.65)	
Varsinais-Suomi	35			1.54 (0.38–6.58)	
Highest education					
Comprehensive	104	0.52 (0.17–1.36)			
Secondary high school or vocational school	195	0.98 (0.56–1.76)	0.004		
Bachelor or university of applied sciences	118	1		1	
Masters of doctorate	70	0.30 (0.12–0.73)			
TBE vaccination status				Not in the model	
Not vaccinated	379	1	Ref		
Vaccinated	102	1.26 (0.69–2.28)	0.44		
Personal history of VBD infection				Not in the model	
No	433	1	Ref		
Yes	46	1.22 (0.53–2.73)	0.64		
History of VBD infection with family and friends				Not in the model	
No	398	1	Ref		
Yes	81	0.77 (0.40–1.45)	0.42		
Language					
Finnish	442	1	Ref	1	
Swedish	49	0.49 (0.14–1.60)	0.25	0.45 (0.12–1.48)	
Activity				Not in the model	
Daily	136	1.14 (0.64–2.03)			
3–4 times a week	141	1			
About once a week	92	1.21 (0.63–2.32)			
A few times a month	40	0.52 (0.17–1.36)	0.33		
About once a month	3	NA			
Less frequently	21	0.85 (0.24–2.66)			
Never	4	0.45 (0.02–4.22)			
Activity varies	51	0.94 (0.43–2.02)			

## Discussion

In this nationwide study on people’s KAP about VBDs, we found several gaps in knowledge and practices that need to be addressed. In addition, we observed some discrepancies between knowledge and practices with participants having a reasonable knowledge but inappropriate practices for TBDs. On the contrary, for MBDs, the knowledge was observed to be inappropriate but practices relatively good.

One important gap detected was the confusion between the characteristics of TBE and LB. Although the respondents generally had good or even excellent knowledge about TBDs, symptoms and protective practices specific to only one of the diseases were mistakenly associated with both. For example, 61% mistakenly agreed on the fact that a ring-like rash symptom is typical for TBE and 80% to the fact that removing tick as fast as possible reduces the risk of TBE. This shows that respondents often confuse the two diseases. Thus, it is important to underline that fact that removing tick as fast as possible is always strongly recommended, especially considering the overlapping risk areas of LB and TBE.

Further, almost 41% thought incorrectly that the available vaccine protects from LB and almost 30% that it protects from tick bites. A previous small-scale KAP study results in highly endemic areas were in line with the findings of this study. However, according to their results, 28% thought that the available vaccine is against LB and 22% that it works against ticks, which indicated that people in these areas are slightly more aware of the TBE vaccine [15]. Our findings might relate to the fact that TBE vaccination is generally addressed as a tick vaccine in public discussion in Finland and promoted as such by certain commercial entities. However, these findings suggest that TBE and LB should be more separated in the public discussion to raise awareness how to use protective measures effectively. The misunderstanding with vaccination may result in people being under false comfort after getting vaccinated and may therefore not use protective measures with proper clothing or perform tick checks and thus, increase the risk for LB. Thus, prevention of tick bites should be highlighted in all communication related to prevention of these diseases.

In addition, we observed a gap related to the risk perception of TBE. The majority of respondents thought that TBE is transmitted through adult ticks and only one third knew that all stages of ticks including larvae and nymph can transmit the disease. Also, majority of the respondents did not know what percentage of ticks in the risk areas carry TBE and more than one fifth thought that the risk is ten times higher than it actually is, as the best current estimate is 1–2% [16, 17]. The respondents tended to think that risk of TBD is much smaller in urban city parks than in the forests, even though many studies have shown that the diversity and prevalence of tick-borne pathogens is comparable between these two types of areas [18, 19].

When asked, where do ticks usually occur, majority of the respondents answered correctly either grass or forest. However, more than six out of ten answered that ticks are commonly found in the skins of furs of animals, and up to 80% of those having pet animals said that they have found a tick from their cat or dog. These findings highlight the One Health approach essential to the effective response to TBDs, as ticks are so frequently encountered through pet animals. We did not assess the protection on pet animals against ticks, but a poll conducted by MSD Animal Health among pet owners showed, that more than 80% of respondents in Finland protect their pet from parasites either all around the year or during summers. In addition, 70% feel that they are sufficiently informed about the dangers of ticks [20]. Our results warrant more detailed research related to ticks and pet owners.

We observed that Finns have generally poor knowledge on MBDs. Only one third of the respondents knew how tularaemia and Pogosta disease are transmitted. In addition, Swedish-speaking respondents were less likely to have a good knowledge on MBDs, which might be explained by the fact that both endemic MBDs in Finland are more prevalent in the areas with mainly Finnish-speaking inhabitants. However, largely MBD-related practice was good or even excellent. This might relate to the fact that Finnish summer is well known for its mosquitoes and as they are causing a lot of nuisance and are visible, people are eager to protect themselves regardless of diseases they could carry. Considering the knowledge related to MBDs, gaps were also detected regarding travel-related MBDs in another study focusing of Finnish travellers and dengue fever [21]. These results indicated that the knowledge regarding MBDs is generally low among Finns as similar gaps were observed in awareness of disease and in the use of protective measures. Based on these results, the awareness needs to be raised both on endemic and tropical MBDs.

Almost all respondents agreed that VBDs can be prevented with right protective measures. However, as 92% (449/488) agreed that LB can be prevented by performing tick checks, only 56% (264/474) reported doing so often or always after outdoor activities. Most used measures were removing ticks as fast as possible, wearing long sleeved clothing, using high pitched boots, and performing tick check after outdoor activities. The proportion that performed tick check often or always is similar to other European studies [22–24]. We found consistency with practices and attitudes to tick repellents, as it was the least used protective measure and only a small share of respondents considered it efficient. Similar observation was done by Zöldi et al. in highly TBE endemic area in Finland. With mosquitoes, the repellents were more used, and they were considered effective [15]. The research related to tick repellents is scarce, and repellents using DEET have been shown to provide protection only in high concentrations [25], which is why tick repellents might be generally considered ineffective. More evidence-based studies are needed to compare the efficacy of repellents for both ticks and mosquitoes to provide better understanding on the topic.

Climate change is strongly linked to VBDs, and we assessed the perception of respondents related to the topic. Almost all the respondents agreed that climate change will influence the prevalence of VBDs and distribution of vector species and 60% expressed their concern of how the distribution of VBDs is altered by climate change. However, when assessing their risk perception, only half felt like they will be more at risk due to climate change and only 39% stated that forecasts of climate change will influence their use of protective measures. This indicates potentially unrealistic optimism and the tendency to assume that bad things are more likely to happen to others than oneself. A poll assessing pet owners’ attitudes towards climate change shows that up to 52% of pet owners think that climate change will increase parasites on pet animals, this finding is in the line with our results regarding risk perception in relation to climate change [20].

Overall, we observed multiple gaps in the knowledge as well as disparities between knowledge and practices. These results clearly call for public health measures, especially through efficient communication. Finnish Institute for Health and Welfare have adapted the results for use, and as being the public health authority, they generally set an example for other authorities to follow. The results have also been communicated to other research institutions that are involved with VBD-related research.

Based on the results, Finnish Institute for Health and Welfare developed communication material that is visible for all citizens in the disease-specific webpages and launched an online survey for citizens to test their knowledge regarding TBDs. In addition, leaflets suitable for both screens and printing were developed, and distributed regarding actions with tick bites and the HDs (welfare counties from 2023 onwards) have been informed about the communication needs. However, sustainable communication strategy is warranted, and it remains important to conduct assessments of the practical effectiveness of the communication strategy and materials.

Furthermore, these results have been used in multiple expert interviews in the media, aiming to correct the misinformation, for example, regarding the vaccine and its effectiveness. However, given the likely increasing trajectory of the incidence of TBDs, currently lacking sustainable financing would be required to maintain awareness raising activities and tackling misinformation.

## Limitations

A few limitations should be considered when interpreting the findings. Based on sample size calculations for simple random sampling, we aimed for a sample of about 3,000 people (confidence level of 95%, expected response rate with 35% and margin of error 2%). However, due to lack of resources, the final sample size had to be reduced to 2,000 and selected by targeted sampling.

The sample size and the response rate were relatively small and may have caused selection bias. As our aim was to enrol participants from all HDs and as the population of Finland is unevenly distributed among the country, we did not sample proportional to the HD population. Thus, there were similar numbers of participants from more densely populated and geographically larger HDs (e.g. the HD of Helsinki and Uusimaa) as well as from sparsely populated and geographically smaller HDs (e.g. HD of Åland). This reduced the national representativeness of the sample, and the study cannot be considered as a representative of the KAP in the general population.

Information bias cannot be ruled out for the following reasons: first, substantially higher proportion of participants were diagnosed with a VBD compared with the proportion of the population diagnosed with VBD. The level of KAP observed among study participants may therefore be higher than in the general population, as persons with a VBD history are more likely to be knowledgeable about the disease. Secondly, the highest response rates were seen in the small island of Åland HD, which is a hyperendemic area for both TBE and LB, potentially influencing the results.

Our study provides an overall assessment of KAP related to VBDs in Finland. KAP surveys are widely used in investigating health behaviours, and they are considered easy to conduct, measure, and interpret [26, 27]. However, one limiting factor relates to KAP study as method, as measuring attitudes via survey has been criticized for people tending to give answers which they believe to be correct and generally acceptable [27, 28].

This study was conducted during COVID-19 pandemic, during which Finnish citizens were encouraged to spend time outdoors instead of indoor gatherings. However, there were no interventions, such as increased communication, towards VBDs. Most likely due to citizens spending more times outdoors, the incidence of TBE in Finland during 2020–2021 rose 1.3 times higher compared to predicted trend [29]. This is unlikely to have had any effects on this study, since even though there were more than usual cases of TBE, there were no additional communication campaigns related to VBDs.

## Conclusion

Our study found gaps in KAP regarding VBDs in Finland. These findings warrant a public health response as there is a link between knowledge and adherence to protective measures [30]. The knowledge regarding TBE vaccination and the separation between TBE and LB in particular needs attention. In addition, general knowledge levels related to both endemic and tropical MBDs needs to be improved. Regarding protective measures against ticks, more adherence to tick inspection and proper clothing is needed, as personal protective measures are essential and often the only means available when dealing with blood-sucking arthropods.

Future research should focus on determining the factors affecting the level of KAP within specific groups of people and aim for build tools for more efficient awareness raising.

## Supporting information

Mäkelä et al. supplementary material 1 Mäkelä et al. supplementary material

Mäkelä et al. supplementary material 2 Mäkelä et al. supplementary material

Mäkelä et al. supplementary material 3 Mäkelä et al. supplementary material

## Data Availability

The data used in this study is available on motivated request to the corresponding author or to Finnish Institute for Health and Welfare.

## Supplementary material

The supplementary material for this article can be found at http://doi.org/10.1017/S0950268824001468.
