# Supplementary material for: Knowledge, attitudes, and practices towards vector-borne diseases in changing climate in Finland
Source: Epidemiol Infect. 2025 Jan 15;153:e12. doi: 10.1017/S0950268824001468 (PMC11748021; doi:10.1017/S0950268824001468)
Supplement: Mäkelä et al. supplementary material 1 — Mäkelä et al. supplementary material [file S0950268824001468sup001.docx]

**Questionnaire**

**Knowledge**

1. **Which of the following diseases have you heard of before?** *You can choose multiple options*
   1. Tick-borne encephalitis
   2. Lyme Borreliosis
   3. Tularaemia
   4. Pogosta disease
2. **What is the cause of tick-borne encephalitis (TBE)?** *Choose one of the options*
   1. Bacteria
   2. Virus
   3. Protozoan
   4. Fungus
   5. Something else, what? _________________________________________
   6. Don´t know
3. **How is Lyme borreliosis transmitted?** *Choose one of the options*
   1. Through a mosquito
   2. Through a tick
   3. Direclty from animals
   4. From human to human
   5. Some other way, how? _________________________________
   6. Don´t know
4. **How is tularaemia mainly transmitted in Finland?** *Choose one of the options*
   1. From human to human
   2. Directly from the animals
   3. Droplet transmission
   4. Through insect bites
   5. Some other way, how? __________________________________
   6. Don´t know
5. **How is Pogosta disease transmitted?** *Choose one of the options*
   1. Through a tick
   2. From human to human
   3. Droplet transmission
   4. Through mosquitoes
   5. Some other way, how? __________________________________
   6. Don´t know
6. **How is borreliosis treated?** *Choose one of the options*
   1. With antibiotics
   2. With anti-inflammatory medication
   3. With specific borreliosis mediciation
   4. With something else, what?_______________________________________
   5. Don´t know
7. **Where do ticks usually occur?** *You can choose multiple options*
   1. In the grass
   2. In the forest
   3. On the skins and furs of animals
   4. Somewhere else, where?______________________________________
   5. Don´t know
8. **Which of the following can transmit tick-borne encephalitis (TBE)?** *You can choose multiple options*
   1. Tick larvae
   2. Nymphs of the ticks
   3. Adults ticks
   4. All above mentioned
   5. Don´t know
9. **What is the approximate proportion of ticks that carry TBE in the risk areas in Finland?** *Choose one of the options*
   1. 1–2 %
   2. 4–8 %
   3. 10–20 %
   4. 40–60 %
   5. Over 90 %
   6. Don´t know
10. **Risk for the tick bite…** *Choose one of the options*
    1. is always high
    2. depents of the environment
    3. is low
    4. there is absolutely no risk
    5. is something else, what?__________________________________

| **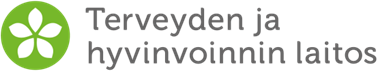**   1. **Choose the correct option** | **Completely disagree** | **Partly disagree** | **Neither disagree nor agree** | **Partly agree** | **Completely agree** |
| --- | --- | --- | --- | --- | --- |
| 1. It is possible to acquire a mosquito-borne disease in Finland |  |  |  |  |  |
| 1. Ticks occur on my municipality of residence |  |  |  |  |  |
| 1. Tick-borne encephalitis (TBE) can require hospital care |  |  |  |  |  |
| 1. Pogosta disease can cause long-term symptoms |  |  |  |  |  |
| 1. There is no treatment available for tularaemia |  |  |  |  |  |
| 1. The risk of acquiring mosquito-borne disease is greater during springs than autumns |  |  |  |  |  |
| 1. A ring-like rash is typical symptom for tick-borne encephalitis (TBE) |  |  |  |  |  |
| 1. Climate change will increase the prevalence of tick-and mosquito-borne diseases in Finland |  |  |  |  |  |
| 1. Climate change will increase the occurence of mosquitoes and ticks |  |  |  |  |  |
| 1. The avaliable vaccine protects against tick bites |  |  |  |  |  |
| 1. The avaliable vaccine protects against tick-borne encephalitis (TBE) |  |  |  |  |  |
| 1. The avaliable vaccine protects from borreliosis |  |  |  |  |  |
| 1. Removing the tick as fast as possible reduces the risk for borreliosis |  |  |  |  |  |
| 1. Removing the tick as fast as possible reduces the risk for tick-borne encephalitis (TBE) |  |  |  |  |  |

**Attitudes related to risks**

| 1. **Choose the option best matching your opinion** | **Completely disagree** | **Partly disagree** | **Neither disagree nor agree** | **Partly agree** | **Completely agree** |
| --- | --- | --- | --- | --- | --- |
| 1. Tick bite almost certainly causes a disease |  |  |  |  |  |
| 1. Mosquito bite will almost certainly cause a disease |  |  |  |  |  |
| 1. I´m concerned of acquiring a tick-borne disease |  |  |  |  |  |
| 1. I´m concerned of acquiring a mosquito-borne disease |  |  |  |  |  |
| 1. Risk of infection caused by ticks is considerably smaller in urban areas (city parks etc) that it is in the forests |  |  |  |  |  |
| 1. I´m at bigger than average risk of aqcuiring TBE infection at my municipality of residence |  |  |  |  |  |
| 1. I´m at bigger than average risk of aqcuiring tularaemia infection at my municipality of residence |  |  |  |  |  |
| 1. I´m at bigger than average risk of aqcuiring pogosta infection at my municipality of residence |  |  |  |  |  |
| 1. I don`t feel like I´m at bigger risk of acquiring a tick- or mosquito-borne disease eventhough the climate would change |  |  |  |  |  |
| 1. I´m concerned of how climate change will affect on distribution of tick-and mosquito-borne diseases |  |  |  |  |  |
| 1. Forecasts of the climate warming and e.i. warmer winters affects on how I protect myself from the insects |  |  |  |  |  |

**Attitudes related to protective measures**

| 1. **Choose the option best matching your opinion**   *NB! In this context, with repellents we mean the repellent used for skin* | **Completely disagree** | **Partly disagree** | **Neither disagree nor agree** | **Partly agree** | **Completely agree** |
| --- | --- | --- | --- | --- | --- |
| 1. It is possible to prevent both tick- and mosquito-borne diseases with right protective measures |  |  |  |  |  |
| 1. You shouldn´t remove the attached tick yourself, but seek a heath care professional to remove it |  |  |  |  |  |
| 1. I trust that the insect repellents are safe for health |  |  |  |  |  |
| 1. The tick repellents used for skin (lotions, sprays etc.) are effective |  |  |  |  |  |
| 1. The mosquito repellents used for skin (lotions, sprays etc.) are effective |  |  |  |  |  |
| 1. Doing a regular tick-check for skin can prevent borreliosis infection |  |  |  |  |  |

**Practices**

1. **If needed, where do you seek information about tick-and mosquito-borne diseases?** *You can choose multiple options*
   1. Newspapers
   2. Wepsite of Finnish Institute of Health and Wellfare (THL)
   3. Somewhere else from the internet, where?___________________________________
   4. Social media (Facebook, Twitter, Instagram..)
   5. Work or school
   6. Vaccination campaigns
   7. Healthcare professional
   8. Friends or family
   9. I trust my own, experience based, knowledge
   10. Somewhere else, where? ____________________________________
2. **Which of the following insect repellents are you using?** *You can choose multiple options*
   1. Skin sprays
   2. Skin lotions
   3. Mosquito smoke
   4. Mosquito repellent that works with gad (i.e. Thermacell)
   5. Something else, what?__________________________________________________________
   6. I don´t use repellent at all

| 1. **How well does the following statements describe your behavior with protecting against ticks and mosquitoes?**   *Choose one of the options (NB! In this context, with repellents we mean the repellent used for skin)* | **Never** | **Rarely** | **Sometimes** | **Often** | **Always or almost always** |
| --- | --- | --- | --- | --- | --- |
| 1. I use tick repellent during outdoor activities in the places where ticks occur |  |  |  |  |  |
| 1. I avoid areas of ticks occurrence |  |  |  |  |  |
| 1. I use mosquito repellents during outdoor activities when mosquitoes are present |  |  |  |  |  |
| 1. I use mosquito hat or other net to protect myself during outdoor activities when mosquitoes are present |  |  |  |  |  |
| 1. I use long sleeved clothes during outdoor activities |  |  |  |  |  |
| 1. I use light colored clothing during outdoor activities |  |  |  |  |  |
| 1. When I´m active in the places of tick occurence, I tuck my trousers bows into my socks |  |  |  |  |  |
| 1. When I´m active in the places of tick occurence, I use high-pitched boots |  |  |  |  |  |
| 1. If I find a tick attached to my skin, I´ll remove it myself as fast as possible |  |  |  |  |  |
| 1. I shower after outdoor activities to get rid of ticks from my skin |  |  |  |  |  |
| 1. I check my skin to detect possible ticks during outdoor activities |  |  |  |  |  |
| 1. After outdoors activities I check my clothes to find possible ticks |  |  |  |  |  |
| 1. I perform a tick-check after outdoor activities |  |  |  |  |  |
| 1. I prefer tick-repellents that are based on natural ingredients |  |  |  |  |  |

**Background**

1. **Have you been vaccinated against tick-borne encephalitis (TBE)?**
   1. Yes. Go to question **18 ja and skip the question 19.**
   2. No. Go to **question 19**
2. **If you answered yes to the question 17, what is the reason for vaccination?** *You can choose multiple options*
   1. I live in the risk area
   2. I own a summerhouse/ spend a lot of time on summerhouse that is located in risk area
   3. I´m very active in the nature in risk areas
   4. Some other reason, what? _______________________________________________
3. **If you answered no to the question 17. what is the reason for not being vaccinated?** *You can choose multiple options*
   1. There is no need for the vaccination, I don´t live in risk areas or I´m not active in nature in risk areas
   2. Vaccination is too expensive
   3. My physician didn´t recommend the vaccine for me
   4. Problems with the vaccination schedule
   5. Some other reason, what? _________________________________________________
4. **Have you or somebody of your friends or family been diagnosed with tick- or mosquito-borne disease?**
   1. I have been diagnosed. With what?_______________________________
   2. My friend or family member have been diagnosed. With what? ____________________________
   3. Have not been diagnosed
5. **Do you own any of the follwin pets?** *Choose one of the options*
   1. Cat
   2. Dog
   3. Both
   4. None of these. Go to the **question 23**.
6. **If you own any of above-mentioned pet animals, have you ever found ticks from their skin or fur?**
   1. Yes
   2. No
7. **If you are active in the nature, what kind of activities you usually engage?** *You can choose multiple options*
8. Walking etc. on marked tracks without stepping aside the road *(asphalt-, gravel- or sawdust roads/tracks)*
9. Walkin etc. on marked tracks with spetting aside the road *(e.g. walking a dog next to tracks or roead in grass or trails)*
10. Walking etc. on own yard
11. Walking etc. in the nature in the neighborhood
12. Walking etc. in the nature in the neighborhood
13. Gardening
14. Hiking or trekking
15. Mushroom picking, berry picking, hunting
16. Walking etc. in the nature with a pet
17. Other nature-related hobby (e.g. orienteering, fishing, frisbeegolfig)
18. Walking etc. in the public beaches
19. Walking etc. in other beach areas
20. A profession that includes being in the nature (e.g. farm work, forest work, research work)
21. Some other activity. What? _________________________________
22. **How often are you active in nature?** *Choose the option that best describes your activity*
    1. 3-4 times a week
    2. About once a week
    3. Few times a month
    4. About once a month
    5. Less frequently
    6. Never
    7. Activity varies. How?___________________________________________________________________
23. **Sex**
    1. Female
    2. Male
    3. Other
24. **Age**

_______years

1. **Monicipality of residence**

______________________

1. **If you own a summer house or send a lot of time in the summer house, where is it located?**

______________________

1. **What is your highest degree?** *Choose the highest level of your education*
2. Primary school
3. Secondary highschool or vocational school
4. Bachelors degree
5. Masters degree
6. Doctorate
7. **If needed, clarify or add details to your answers here**

__________________________________________________________________________________________________________________________________________________________________________________________________________________________________________________________________________________________________________________________________________________________________________________________________________________________________________________________________________________________________________________________________________________________________________________________________________________________________________________________________________________________________________________________________________________________________________________________________________________

**Thank you for your response!**

**!**
