# Supplementary material for: Knowledge, attitudes, and practices towards vector-borne diseases in changing climate in Finland
Source: Epidemiol Infect. 2025 Jan 15;153:e12. doi: 10.1017/S0950268824001468 (PMC11748021; doi:10.1017/S0950268824001468)
Supplement: Mäkelä et al. supplementary material 2 — Mäkelä et al. supplementary material [file S0950268824001468sup002.docx]

| Practices | **Points rewarded/ more detailed scoring** | | |  |  |
| --- | --- | --- | --- | --- | --- |
| 17 | Never | Rarely | Sometimes | Often | Always or almost always |
| 1. I use tick repellent during outdoor activities in the places where ticks occur | 0 | 0 | 1 | 1 | 2 |
| 2. I avoid areas of ticks occurrence | NA | NA | NA | NA | NA |
| 3. I use mosquito repellents during outdoor activities when mosquitoes are present | 0 | 0 | 1 | 1 | 2 |
| 4. I use mosquito hat or other net to protect myself during outdoor activities when mosquitoes are present | 0 | 0 | 1 | 1 | 2 |
| 5. I use long sleeved clothes during outdoor activities | 0 | 0 | 1 | 1 | 2 |
| 6. I use light colored clothing during outdoor activities | 0 | 0 | 1 | 1 | 2 |
| 7. When I´m active in the places of tick occurence, I tuck my trousers bows into my socks | 0 | 0 | 1 | 1 | 2 |
| 8. When I´m active in the places of tick occurence, I use high-pitched boots | 0 | 0 | 1 | 1 | 2 |
| 9. If I find a tick attached to my skin, I´ll remove it myself as fast as possible | 0 | 0 | 1 | 1 | 2 |
| 10. I shower after outdoor activities to get rid of ticks from my skin | NA | NA | NA | NA | NA |
| 11. I check my skin to detect possible ticks during outdoor activities | NA | NA | NA | NA | NA |
| 12. After outdoors activities I check my clothes to find possible ticks | 0 | 0 | 1 | 1 | 2 |
| 13. I perform a tick-check after outdoor activities | 0 | 0 | 1 | 1 | 2 |
| 14. I prefer tick-repellents that are based on natural ingredients | NA | NA | NA | NA | NA |
