# Supplementary material for: Knowledge, attitudes, and practices towards vector-borne diseases in changing climate in Finland
Source: Epidemiol Infect. 2025 Jan 15;153:e12. doi: 10.1017/S0950268824001468 (PMC11748021; doi:10.1017/S0950268824001468)
Supplement: Mäkelä et al. supplementary material 3 — Mäkelä et al. supplementary material [file S0950268824001468sup003.docx]

**Supplementary table 1.** Univariate and multivariable analysis of factors associated with good attitudes regarding mosquito-borne diseases.

**Supplementary table 2.** Univariate and multivariable analysis of factors associated with good practices regarding mosquito-borne diseases.

**Supplementary table 3.** Univariate and multivariable analysis of factors associated with good knowledge regarding tick-borne diseases.

**Supplementary table 4.** Univariate and multivariable analysis of factors associated with good attitudes regarding tick-borne diseases.
